# Maternal overweight and obesity and the risk of caesarean birth in Malawi

**DOI:** 10.1186/s12978-019-0700-2

**Published:** 2019-04-03

**Authors:** Owen Nkoka, Peter Austin Morton Ntenda, Thomas Senghore, Paul Bass

**Affiliations:** 1 School of Public Health, College of Public Health, Taipei Medical University, Taiwan No. 250, Wu-Hsing Street, Taipei, 110 Taiwan; 2 School of Public Health and Family Medicine, Department of Public Health, College of Medicine, University of Malawi, Private Bag 360, Chichiri, Blantyre, 3 Malawi; 3 Department of Public & Environmental Health, School of Medicine & Allied Health Sciences, University of The Gambia, P. O. Box 3530, Brikama, The Gambia; 4 Department of Nursing and Reproductive Health, School of Medicine & Allied Health Sciences, University of The Gambia, P. O. Box 3530, Banjul Campus, The Gambia

**Keywords:** Body mass index, Overweight, Obesity, Caesarean births, Malawi

## Abstract

**Background:**

Overweight and obese women are at risk of pregnancy and delivery complications. This study investigates the trend and association between maternal overweight and obesity on caesarean births in Malawi.

**Methods:**

We utilised cross-sectional population-based Demographic Health Surveys (DHSs) data collected from mothers aged 18–49 years in 2004/05, 2010, and 2015/16 in Malawi. The outcome measure was caesarian birth within 5 years preceding the surveys. The main independent variable was maternal Body Mass Index (BMI) measured as weight in kilograms by height in meters squared (kg/m^2^) and categorized according to the World Health Organization (WHO) guidelines. Generalized estimating equations (GEE) regression models were constructed to analyze total samples of 6795, 4474 and 4363 in 2004/05, 2010 and 2015/16 respectively.

**Results:**

There was an observed increase in the trend of caesarean births as well as maternal overweight and obesity from 2004 to 2015. The results of the multivariate analyses showed that maternal overweight (adjusted odds ratio [aOR] = 1.35; 95% Confidence Interval [CI] 1.01–1.83) in 2015/16 and (aOR = 1.36; 95% CI: 1.10–1.65) from 2004 to 2015 were risk factors for caesarean births in Malawi. In addition, being obese (aOR = 2.15; 95% CI: 1.12–4.11) in 2004/05, (aOR = 1.66; 95% CI: 1.08–2.55) in 2010, (aOR = 2.18; 95% CI: 1.48–3.21) in 2015/16, and (aOR = 2.16; 95% CI: 1.65–2.84) from 2004 to 2015) increased the risk of caesarean births. In addition, women who had one parity, and lived in the northern region were significantly more likely to have undergone caesarean birth.

**Conclusions:**

In order to reduce non-elective cesarean birth in Malawi, specific public health programs should be focus on reducing overweight and obesity among women of reproductive age. More focus attention may be given to women with one parity, particularly in the urban and the northern region of Malawi.

## Plain English summary

Women who are overweight or obese have difficulties during pregnancy and delivery. Using Malawi Demographic Health Survey (MDHS) data collated in 2004/05, 2010 and 2015/16, this study determined the trend and association between women who were overweight or obese and caesarean birth in Malawi.

Study participants were women aged 15–49 years who had given birth through caesarean section (CS) within 5 years preceding the surveys. Overweight and obesity as the main independent variables were measured as weight in kilograms by height in meters squared. Of 764 participating women who had delivered through CS and included in this analysis, 237 in 2004/05, 225 in 2010 and 302 in 2015/16 respectively delivered through CS in Malawi.

The results showed an increasing trend in CS, overweight and obesity among women from 2004 to 2015. In addition, overweight and obese women were associated with increased risk of CS. Furthermore, women with parity or live in the urban and northern region of Malawi are significantly more likely to have undergone caesarean birth.

In conclusion, in order to prevent or reduce CS in Malawi, specific public health programs should be focus on the reduction of overweight and obesity among women of reproductive age. Women with one parity should be prioritized, particularly in the urban and the northern region of Malawi.

## Background

Overweight and obesity are a major public health concern contributing to more than 2 million preventable deaths each year [1, 2]. According to the World Health Organization (WHO), overweight is defined as body mass index (BMI) of ≥25 kg/m^2^ and obesity as BMI ≥ 30 kg/m^2^ [3]. In certain countries in Africa, up to 50% of the populations are classified as either overweight or obese [4, 5] and has been attributed to changes in lifestyle, cultural and environmental factors [6]. Overweight and obesity in African society is often viewed as a sign of social status, wellness and prosperity [7–9] particularly among women. Previous studies have shown that African women are 4 -times more likely to be obese than those of their male counterparts [10]. In Malawi, one in five (21%) women aged 15–49 years are overweight or obese [11], similar to 20.9% reported in Nigeria [9].

Although overweight and obesity are known risk factors for many health problems, including cardiovascular diseases [2, 6], women who are overweight or obese also have an increased risk of complications during pregnancy and delivery [12, 13]. Previous studies have shown that overweight and obese women had increased risk of maternal and fetal complications such as gestational diabetes, hypertension, fetal distress, preeclampsia, postpartum haemorrhage, genital tract infection, intrauterine death and macrosomia which are known to increase the risk for caesarean birth [14–18]. These complications can result in disabilities or deaths particularly in settings with limited resources and capacity to properly conduct safe surgery or manage surgical complications [19].

In Malawi, the rates of caesarean births have doubled in the last decades (from 3% in 1992 to 6% in 2016) [11]. Studies have linked the mother’s body composition with birth weight and other related infant health outcomes [20, 21]. The risks following caesarean births include placenta accrete, hysterectomy and a high proportion of uterine scars that may cause further complications in subsequent pregnancies [22]. However, population-based studies linking overweight and obesity to caesarean births in Africa are limited. Therefore, understanding the relationship between overweight and obesity to caesarean births is important for designing effective interventions to prevent or reduce the incidence of caesarean births in Malawi or Africa. Accordingly, the objectives of this paper were to (i) explore the trends in maternal overweight, obesity, and caesarean birth and (ii) to investigate whether maternal overweight and obesity are risk factors for caesarean births in Malawi using population-based survey data.

## Methods

### Study design and sampling technique

This was a cross-sectional study and used 2004/05, 2010 and 2015/16 Malawian Demographic and Health Survey (MDHS) data, from a nationally representative sample. The methods used in this study have been described in detail elsewhere [11, 23, 24]. In brief, a stratified two-stage cluster design in the MDHS was used to produce a nationally representative sample of women of reproductive age (15–49 years). The sampling frame used for the 2004/05, and 2010 and 2015/16 MDHSs were drawn from the Malawi population and housing censuses (MPHC) of 1998 and 2008 respectively. In the first stage, 522, 849 and 850 clusters and in the second stage 15,091, 27,345, and 27,516 households in 2004/05, 2010, and 2015/16 were selected respectively. We restricted samples to those women with most recent births within the last 5 years before the surveys, and the analyses were limited to mothers who had measurements for height and weight.

### Data collection

Face-to-face interviews were used to collect information from women aged 15–49 years with children below the age of 5 years prior to the surveys. Information on sociodemographic, environmental, immunization, household characteristics, anthropometric, and infant and young child health care indicators were collected by trained enumerators. To avoid confounding, we restricted our sample to women aged 18–49 [25]. Following standard procedures, anthropometric measurements of height and weight were collected for women. Height was measured using standardized measuring boards with accuracy to 0.1 cm while weight was measured using solar-powered scales with an accuracy of 0.1 kg [11, 23, 24].

### Measures

#### Variables

The outcome variable was caesarian birth within 5 years preceding the surveys and the main independent variable was maternal BMI measured as weight in kilograms by height in meters squared (kg/m^2^) based on WHO conventional classification and was categorized into four groups (underweight < 18.5, normal 18.5–24.9, overweight 25.0–29.9 kg/m^2^ and obesity ≥30.0 kg/m^2^) [3, 4].

#### Covariates

We included sex of the child, maternal age (years), maternal educational level, household wealth, parity, number of antenatal care (ANC) visits, tobacco smoking, place of residence and geographical region. Sex of the child was categorized as male or female, while maternal age was categorised into five groups (18–19, 20–24, 25–29, 30–34, and 35–49). Maternal education was categorized as no formal education, primary education, and secondary education and above. ANC visit was grouped as adequate or inadequate as per WHO recommendations [26]. Mothers with four and above ANC visits were categorized as having adequate care, while those with less than four ANC visits were regarded as having inadequate care. Parity was defined as the number of children previously born in the household. However, for the purpose of this study, we created four categories so as to fit our analysis. The groups were one or none, two to three children, four to five children and six children and above. Maternal smoking status was assessed whether a mother smoked tobacco or not. We included two variables to represent an area of residence as the place of residence and geographical region. Place of residence was categorized as urban or rural area, whilst geographical region was grouped as northern, central, and southern regions. The household wealth index was categorized in quintiles (poorest, poor, middle, rich, and richest). The wealth index is a composite measure of a household’s cumulative living standard and was calculated using easy-to-collect data on a household’s ownership of selected assets, such as televisions and bicycles, materials used for constructing the house, access to safe drinking water, access to improved sanitation facilities, and other characteristics of a household. Household asset scores were generated through a principal component analysis [11, 23, 24].

### Statistical analyses

Analyses were conducted separately for 2004, 2010, 2015, and between 2004 and 2015 respectively. Descriptive and bivariate analyses were performed to describe the main variables and the relationship between explanatory factors and caesarean birth. In addition, the Cochran-Armitage Trend Test was used to assess trend prevalence in maternal overweight and obesity on caesarean birth across the three cohort years (2004 to 2015/16). Owing to the nature of MDHS sampling design, survey-specific SAS procedures for weighting, clustering, and stratification in the survey designs were used where appropriate. PROC SURVEYFREQ procedure was used to estimate the weighted prevalence of caesarean birth in mothers aged 18–49 years in the total population as well as within subgroups of the population. Frequency, percentages, and standard errors were presented to describe the sample characteristics.

Using PROC GENMOD in SAS, the multivariate analyses were conducted fitting a series of logistic models with generalized estimating equations (GEE) for estimating the effects of maternal BMI on the risk of caesarean birth while controlling for other confounding factors. The GEE model assumes a binomial distribution with a logit link functions on the probability of parameters. Thus, owing to the nature of MDHS complex data structure, the GEE models were used to adjust the correlated individual responses. The results of the multivariate analysis were obtained using adjusted odds ratios (aORs) with their *p*-values and 95% confidence intervals (CIs). The significance level of alpha was set at 5%. The variance inflation factor (VIF) was used for assessing multicollinearity in the model. Multicollinearity was not detected because the VIF values were less than 10. All analyses were performed using SAS software version 9.4 (SAS Institute Inc., Cary, NC, USA).

### Ethics statement

The protocol for sample collection and the questionnaires were reviewed and approved by the Malawi National Health Sciences Research Committee, the Institutional Review Board of ICF Macro, and the Centers for Disease Control in Atlanta. Informed consent was obtained at the beginning of each interview and the authors sought permission from the DHS program for the use of the data.

## Results

**Fig. 1:**
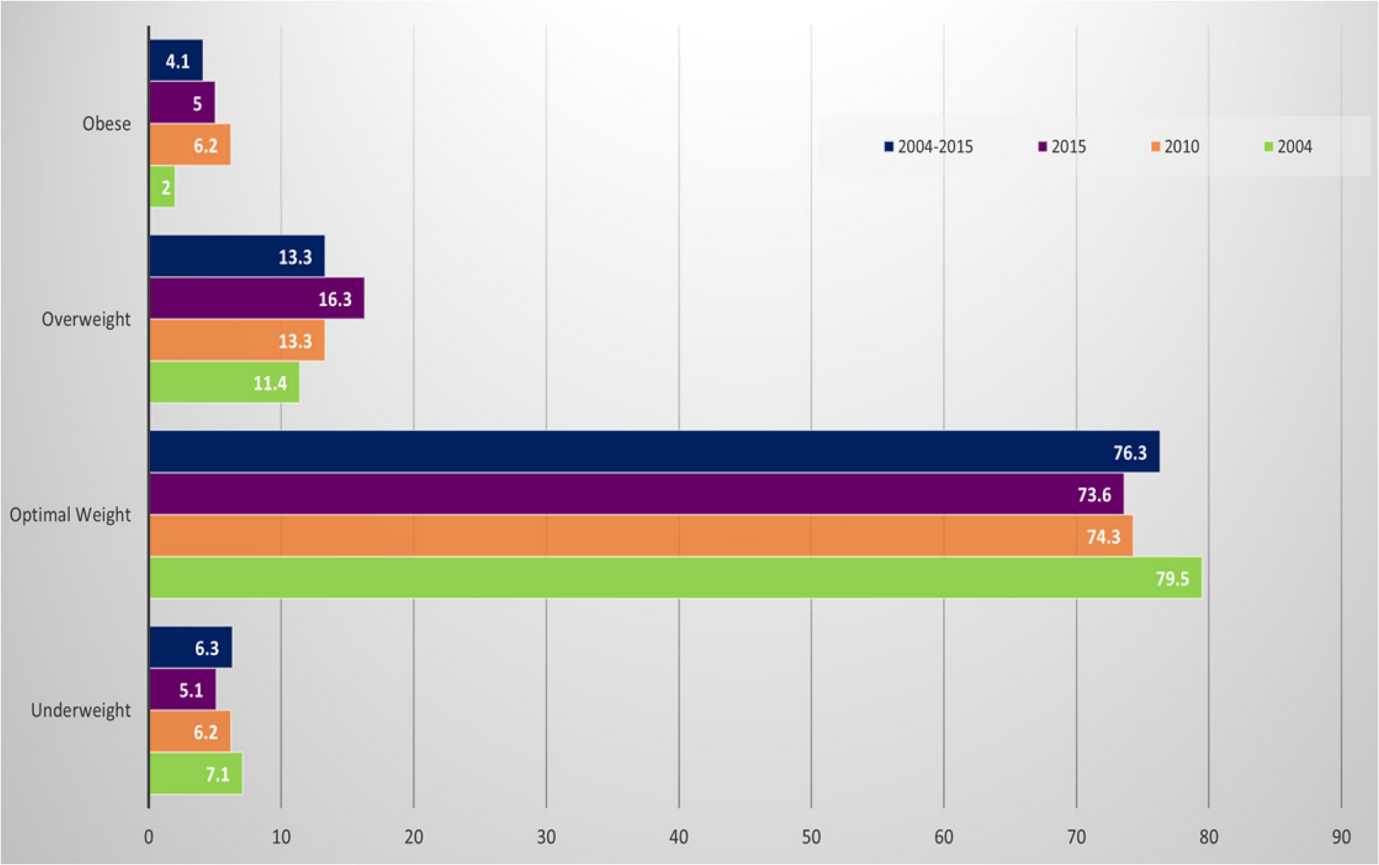
Trends of maternal body mass index between 2004 and 2015

The prevalence of maternal overweight was 11, 13, 16, and 13% in 2004/05, 2010, 2015/16 and 2004–2015 respectively, while maternal obesity was 2, 6, 5 and 4% in 2004/05, 2010, 2015/16, and 2004–2015 respectively (Fig. 1). Table 1 presents the baseline characteristics of the study population. Across all the three cohort years, more than two-thirds of mothers had normal BMI (80%) in 2004/05, (74%) in 2010 as well as 2015/16 and (76%) for 2004–2015. In all cohort years, the majority of the women were aged 20–24 years, had at least primary education, had 2–3 children, were non-smokers and lived in the rural areas. However, except in 2010, over 50% of women in other cohort years had adequate ANC.

**Table 1:** Baseline Characteristics of the Study Population

Characteristics	2004			2010			2015			2004—2015		
	*N* = 6795			*N* = 4474			*N* = 4363			*N* = 15,732		
	*n*	(%)	SE	*n*	(%)	SE	*n*	(%)	SE	*n*	(%)	SE
Mode of delivery												
Vaginal delivery	6558	(96.5)	0.3	4349	(95.3)	0.4	4061	(93.8)	0.5	14,968	(95.4)	0.2
Caesarian delivery	237	(3.5)	0.3	225	(4.7)	0.4	302	(6.2)	0.5	764	(4.6)	0.2
BMI category (kg/m)												
Underweight (< 18.5)	485	(7.1)	0.4	272	(6.2)	0.5	224	(5.1)	0.4	981	(6.3)	0.3
Normal (18.5–24.9)	5432	(79.5)	0.6	3424	(74.3)	0.9	3174	(73.6)	0.9	12,030	(76.3)	0.5
Overweight (25.0–29.9)	743	(11.4)	0.5	599	(13.3)	0.6	736	(16.3)	0.7	2078	(13.3)	0.4
Obese (≥30)	135	(2.0)	2.0	279	(6.2)	0.5	229	(5.0)	0.4	643	(4.1)	0.2
Sex of the child												
Male	3485	(51.1)	0.7	2295	(49.7)	1.0	2176	(49.4)	0.9	7956	(50.2)	0.5
Female	3310	(48.9)	0.7	2279	(50.3)	1.0	2187	(50.6)	0.9	7776	(49.8)	0.5
Maternal age (years)												
15–19	462	(6.8)	0.4	260	(5.7)	0.4	281	(6.8)	0.5	1003	(6.5)	0.3
20–24	2196	(32.5)	0.7	1225	(27.2)	0.8	1283	(29.4)	0.9	4704	(30.1)	0.5
25–29	1735	(25.8)	0.6	1307	(29.4)	0.9	1094	(24.6)	0.8	4136	(26.5)	0.4
30–34	1100	(16.0)	0.5	829	(17.2)	0.7	856	(20.1)	0.8	2785	(17.5)	0.4
35–49	1302	(18.9)	0.6	953	(20.5)	0.8	849	(19.2)	0.7	3104	(19.4)	0.4
Mother’s educational level												
No formal education	1732	(24.9)	0.9	750	(16.8)	0.8	525	(12.7)	0.6	3007	(19.2)	0.5
Primary	4288	(63.1)	0.8	3131	(66.9)	0.9	2779	(65.6)	0.9	10,198	(64.6)	0.5
Secondary or higher	775	(12.0)	0.6	693	(16.3)	0.8	1059	(22.7)	0.8	2527	(16.2)	0.5
Household wealth												
Poorest	1292	(18.9)	0.7	967	(19.7)	0.9	918	(23.5)	0.8	3177	(20.5)	0.5
Poor	1493	(21.3)	0.8	989	(21.5)	0.8	927	(22.1)	0.9	3409	(21.6)	0.5
Middle	1584	(22.5)	0.7	1029	(21.1)	0.8	850	(19.1)	0.7	3463	(21.1)	0.4
Richer	1374	(20.1)	0.7	903	(18.5)	0.7	820	(18.0)	0.8	3097	(19.1)	0.4
Richest	1052	(17.1)	1.2	686	(19.1)	1.1	848	(17.3)	0.7	2586	(17.8)	0.7
Parity												
1	1263	(19.3)	0.6	706	(16.3)	0.6	965	(22.2)	0.8	2934	(19.2)	0.4
2–3	2487	(37.0)	0.7	1668	(40.0)	0.9	1731	(39.5)	1.0	5886	(38.0)	0.5
4–5	1609	(23.0)	0.6	1212	(25.7)	0.8	1010	(23.3)	0.8	3831	(23.9)	0.4
≥ 6	1436	(20.7)	0.6	988	(20.0)	0.7	657	(14.9)	0.7	3081	(18.9)	0.4
Number of ANC visits												
< 4	2839	(41.8)	0.8	2540	(54.2)	0.9	2125	(48.3)	1.0	7504	(47.3)	0.5
≥ 4	3956	(58.2)	0.8	2034	(45.8)	0.9	2238	(51.7)	1.0	8228	(52.7)	0.5
Smoking												
No	6776	(99.8)	0.1	4554	(99.5)	0.1	4337	(99.5)	0.1	15,667	(99.6)	0.1
Yes	19	(0.2)	0.1	20	(0.5)	0.1	26	(0.5)	0.1	65	(0.4)	0.1
Place of residence												
Urban	761	(13.8)	1.5	489	(16.0)	1.0	763	(14.3)	0.6	2013	(14.6)	0.9
Rural	6034	(86.2)	1.5	4085	(84.0)	1.0	3600	(85.7)	0.6	13,719	(85.4)	0.9
Geographical region												
Northern	897	(13.2)	1.1	798	(11.2)	0.7	797	(11.2)	0.4	2492	(12.1)	0.7
Central	2486	(39.5)	1.7	1591	(43.8)	1.2	1534	(43.2)	0.8	5611	(41.8)	1.0
Southern	3412	(47.3)	1.7	2185	(45.0)	1.1	2032	(45.6)	0.8	7629	(46.1)	1.0

*ANC* Antenatal Care, *BMI* Body Mass Index, *SE* Standard Error, *%* are weighted to reflect population characteristics of children and their mothers at the national levels; *n* are unweighted sample sizes

Tables 2 present bivariate analyses of sociodemographic factors and caesarean birth and overweight and obesity on caesarean trend prevalence across the three cohort years. There was an observed increase in the trend for maternal overweight and obesity on caesarean birth across cohort years (*p* < 0.001). Additionally, obese mothers were more likely to have a caesarean birth. Furthermore, in all cohort years, women who had secondary education and above, from richest households, with one child, adequate ANC, resided in urban area and central region were more likely to have increased risk of caesarean birth.

**Table 2:** Bivariate analysis of sociodemographic and delivery factors and trends in maternal overweight and obesity by caesarian births from 2004 to 2015

Characteristics	2004		2010		2015		2004–2015	
	No	Yes	No	Yes	No	Yes	No	Yes
	*n* (%)	*n* (%)	*n* (%)	*n* (%)	*n (%)*	*n (%)*	*n (%)*	*n (%)*
BMI category (kg/m)								
Underweight (< 18.5)	469 (96.7)	16 (3.3)^c^	265 (97.4)	7 (2.6)^b^	214 (95.5)	10 (4.5)^a^	11,509 (95.7)	521 (4.3)
Normal (18.5–24.9)	5253 (96.7)	179 (3.3)	3266 (95.4)	158 (4.6)	2990 (94.2)	184 (5.8)	1941 (93.4)	137 (6.6)
Overweight (25.0–29.9)	712 (95.8)	**31 (4.2)**	563 (94.0)	**36 (6.0)**	666 (90.5)	**70 (9.5)†**	570 (88.7)	73 (11.3)
Obese (≥30)	124 (91.9)	**11 (8.1)**	255 (91.4)	**24 (9.6)**	191 (83.4)	**38 (16.6)†**	11,509 (95.7)	521 (4.3)
Sex of the child								
Male	3359 (96.4)	126 (3.6)	2176 (94.8)	119 (5.2)	2014 (92.6)	162 (7.4)	7549 (94.9)	407 (5.1)
Female	3199 (96.7)	111 (3.3)	2173 (95.4)	106 (4.6)	2047 (93.6)	140 (6.4)	7419 (95.4)	357 (4.6)
Maternal age (years)								
15–19	434 (93.9)	28 (6.1)^b^	248 (95.4)	12 (4.6)^c^	264 (94.0)	17 (6.0)^c^	946 (94.3)	57 (5.7)^b^
20–24	2112 (96.2)	84 (3.8)	1153 (94.1)	72 (5.9)	1198 (93.4)	85 (6.6)	4463 (94.9)	241 (5.1)
25–29	1676 (96.6)	59 (3.4)	1234 (94.4)	73 (5.6)	1000 (91.4)	94 (8.6)	3910 (94.5)	226 (5.5)
30–34	1066 (96.9)	34 (3.1)	803 (96.9)	26 (3.1)	792 (92.5)	64 (7.5)	2661 (95.5)	124 (4.5)
35–49	1270 (97.5)	32 (2.5)	911 (95.6)	42 (4.4)	807 (95.1)	42 (4.9)	2988 (96.3)	116 (3.7)
Mother’s educational level								
No formal education	1695 (97.9)	37 (2.1)^a^	732 (97.6)	18 (2.4)^a^	508 (96.8)	17 (3.2)^a^	2935 (97.6)	72 (2.4)^a^
Primary	4147 (96.7)	141 (3.3)	2994 (95.6)	137 (4.4)	2622 (94.4)	157 (5.6)	9763 (95.7)	435 (4.3)
Secondary or higher	716 (92.4)	59 (7.6)	623 (89.9)	70 (10.1)	931 (87.9)	128 12.1)	2270 (89.8)	257 (10.2)
Household wealth								
Poorest	1249 (96.7)	43 (3.3)^a^	933 (96.5)	34 (3.5)^a^	879 (95.8)	39 4.2)^a^	3061 (96.4)	116 (3.6)^a^
Poor	1454 (97.4)	39 (2.6)	953 (96.4)	36 (3.6)	892 (96.2)	35 (3.8)	3299 (96.8)	110 (3.2)
Middle	1544 (97.5)	40 (2.5)	990 (96.2)	39 (3.8)	802 (94.4)	48 (5.6)	3336 (96.3)	127 (3.7)
Richer	1342 (96.4)	50 (3.6)	853 (94.5)	50 (5.5)	763 (93.1)	57 (6.9)	2940 (94.9)	157 (5.1)
Richest	987 (93.8)	65 (6.2)	620 (90.4)	66 (9.6)	725 (85.5)	123(14.5)	2332 (90.2)	254 (9.8)
Parity								
1	1184 (93.8)	79 (6.2)^a^	640 (90.7)	66 (9.3)^a^	871 (90.3)	94 (9.7)^a^	2695 (91.9)	239 (8.1)^a^
2–3	2409 (96.9)	78 (3.1)	1582 (94.8)	86 (5.2)	1599(92.4)	132 (7.6)	5590 (95.0)	296 (5.0)
4–5	1564 (97.2)	45 (2.8)	1174 (96.9)	38 (4.1)	959 (95.0)	51 (4.5)	3697 (96.5)	134 (3.5)
≥ 6	1401 (97.6)	35 (2.4)	953 (96.5)	35 (3.5)	632 (96.2)	25 (3.8)	2986 (96.9)	95 (3.1)
Number of ANC visits								
< 4	2774 (97.7)	65 (2.3)^b^	2434 (95.8)	106(4.2)^b^	2009(94.5)	116 5.5)^b^	7217 (96.2)	287 (3.8)^a^
≥ 4	3784 (95.7)	172 (4.3)	1915 (94.2)	119 (5.8)	2052(91.7)	186 (8.3)	7751 (94.2)	477 (5.8)
Smoking								
No	6540 (96.5)	236 (3.5)	4330 (95.1)	224 (4.9)	4036(93.1)	301 (6.9)	14,906 (95.1)	761 (4.9)
Yes	18 (94.7)	1 (5.3)	19 (95.0)	1 (5.0)	25 (96.2)	1 (3.8)	62 (95.4)	3 (4.6)
Place of residence								
Urban	716 (94.1)	45 (5.9)^b^	449 (91.8)	40 (8.2)^b^	658 (86.2)	105 (13.8)^a^	1823 (90.6)	190 (9.4)^a^
Rural	5842 (96.8)	192 (3.2)	3900 (95.5)	185 (4.5)	3403(94.5)	197 (5.5)	13,145 (95.8)	574 (4.2)
Geographical region								
Northern	852 (95.0)	47 (4.5)^c^	752 (94.2)	46 (5.8)	710 (89.1)	7(10.9)^a^	2314 (92.9)	178 (7.1)^a^
Central	2409 (96.9)	77 (3.1)	1521 (95.6)	70 (4.4)	1433(93.4)	101 (6.6)	5363 (95.6)	248 (4.4)
Southern	3297 (96.6)	115 (3.4)	2076 (95.0)	109 (5.0)	1918(94.4)	114 (5.6)	7291 (95.6)	338 (4.4)

*ANC* Antenatal Care, *BMI* Body Mass Index; ^*a*^*P* < 0.0001; ^*b*^*P* < 0.001; ^*c*^*P* < 0.05; †*p*-trend, < 0.001

Boldface shows results of the trend analysis that overweight and obesity and caesarean births significantly increased by cohort years from 2004 to 2015

**Fig. 2:**
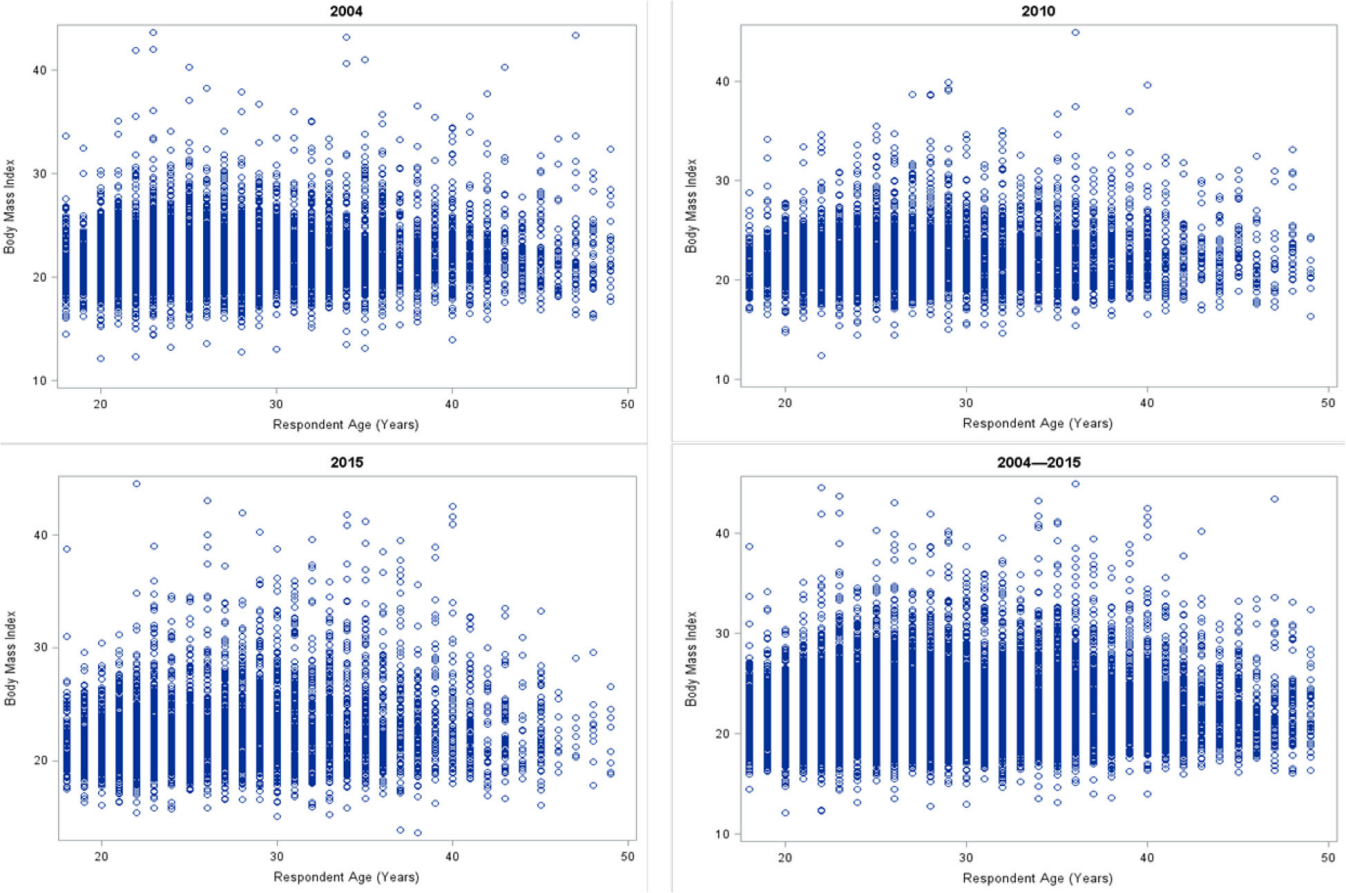
Scatter plots of respondents’ age and Body Mass Index in 2004, 2010, 2015 and 2004–2015

Figure 2 shows scatter plots for maternal BMI by maternal age in 2004, 2010, 2015, and 2004–2015. In all the cohort years, there was a positive correlation between maternal BMI with an increase in age. Overall, the patterns of maternal BMI and age had similar distributions across all the cohort years.

Table 3 presents the multivariate logistic regression results. After controlling for potential confounders (sex of the child, maternal age, educational level, household wealth, parity, number of ANC visits, smoking, place of residence, and geographical region), compared to normal BMI, overweight mothers had increased odds of caesarian birth (adjusted odds ratio [aOR] = 1.36; 95% Confidence Interval [CI] 1.01–1.83) in 2015/16 and (aOR = 1.35; 95% CI: 1.10–1.65) from 2004 to 2015. In addition, obese women (aOR = 2.15; 95% CI: 1.12–4.11) in 2004/05, (aOR = 1.66; 95% CI: 1.08–2.55) in 2010, (aOR = 2.18; 95% CI: 1.48–3.21) in 2015/16, and (aOR = 2.16; 95% CI: 1.65–2.84) from 2004 to 2015) had increased the risk of caesarean births. In terms of covariates included in this study, mothers with no formal and primary education had reduced odds of caesarean birth in 2004/05, 2010, and between 2004 and 2015 compared to mothers with secondary education and above. Compared to mothers from richest households, mothers from poor households had reduced odds of caesarean birth in 2010, 2015/16, and 2004–2015. Mothers with one child had increased odds of caesarean birth compared to mothers with 6 children or more in 2010, 2015 and 2004–2015. Mothers with inadequate ANC visits had reduced odds of caesarian birth compared to their counterparts in 2015 and 2004–2015. Furthermore, the odds of caesarean birth were increased in mothers who resided in the northern region compared to the southern region dwellers.

**Table 3:** Multivariate Logistic Regression for Maternal Obesity and the Risk of Caesarian Section

Characteristics	2004		2010		2015		2004—2015	
	AORs	95% (CI)	AORs	95% (CI)	AORs	95% (CI)	AORs	95% (CI)
BMI category (kg/m2)								
Underweight (< 18.5)	1.04	(0.61–1.76)	0.53	(0.24–1.15)	0.77	(0.38–1.55)	0.78	(0.54–1.12)
Normal (18.5–24.9)	1.00	Ref.	1.00	Ref.	1.00	Ref.	1.00	Ref.
Overweight (25.0–29.9)	1.25	(0.83–1.87)	1.19	(0.80–1.77)	**1.36**	**(1.01–1.83)** ^**c**^	**1.35**	**(1.10–1.65)** ^**b**^
Obese (≥30)	**2.15**	**(1.12–4.11)** ^**c**^	**1.66**	**(1.08–2.55)** ^**c**^	**2.18**	**(1.48–3.21)** ^**a**^	**2.16**	**(1.65–2.84)** ^**a**^
Sex of the child								
Male	1.10	(0.84–1.43)	1.11	(0.84–1.48)	1.19	(0.93–1.51)	1.13	(0.97–1.30)
Female	1.00	Ref.	1.00	Ref.	1.00	Ref.	1.00	Ref.
Maternal age (years)								
18–19	1.37	(0.61–3.08)	**0.28**	**(0.11–0.70)** ^**b**^	0.57	(0.26–1.22)	**0.55**	**(0.35–0.87)** ^**c**^
20–24	1.03	(0.51–2.09)	0.53	(0.27–1.02)	0.66	(0.37–1.19)	**0.61**	**(0.42–0.88)** ^**b**^
25–29	1.15	(0.63–2.11)	0.71	(0.39–1.30)	0.99	(0.58–1.67)	0.86	(0.62–1.19)
30–34	1.27	(0.74–2.17)	**0.55**	**(0.32–0.95)** ^**c**^	1.07	(0.67–1.73)	0.94	(0.70–1.27)
35–49	1.00	Ref.	1.00	Ref.	1.00	Ref.	1.00	Ref.
Mother’s educational level								
No formal education	**0.48**	**(0.29–0.81)** ^**b**^	**0.41**	**(0.22–0.77)** ^**b**^	0.67	(0.38–1.17)	**0.47**	**(0.35–0.64)** ^**a**^
Primary	**0.61**	**(0.42–0.89)** ^**c**^	**0.67**	**(0.47–0.96)** ^**c**^	0.87	(0.65–1.17)	**0.69**	**(0.57–0.83)** ^**b**^
Secondary or higher	1.00	Ref.	1.00	Ref.	1.00	Ref.	1.00	Ref.
Household wealth								
Poorest	0.92	(0.55–1.52)	**0.60**	**(0.36–0.99)** ^**c**^	**0.57**	**(0.36–0.91)** ^**c**^	**0.72**	**(0.54–0.94)** ^**c**^
Poor	0.71	(0.42–1.19)	**0.55**	**(0.35–0.92)** ^**c**^	**0.47**	**(0.30–0.73)** ^**c**^	**0.59**	**(0.45–0.78)** ^**b**^
Middle	0.65	(0.40–1.06)	**0.55**	**(0.34–0.87)** ^**c**^	**0.66**	**(0.45–0.98)** ^**c**^	**0.63**	**(0.49–0.82)** ^**b**^
Richer	0.82	(0.52–1.28)	0.74	(0.50–1.10)	0.71	(0.50–1.00)	**0.77**	**(0.61–0.96)** ^**c**^
Richest	1.00	Ref.	1.00	Ref.	1.00	Ref.	1.00	Ref.
Parity								
1	1.98	(0.91–4.12)	**3.71**	**(1.76–7.83)** ^**b**^	**3.11**	**(1.51–6.43)** ^**b**^	**3.18**	**(2.10–4.81)** ^**a**^
2–3	1.10	(0.57–2.09)	1.65	(0.86–3.17)	1.87	(0.97–3.58)	**1.70**	**(1.19–2.42)** ^**b**^
4–5	1.04	(0.61–1.73)	0.96	(0.54–1.70)	1.11	(0.63–1.98)	1.09	(0.80–1.49)
≥ 6	1.00	Ref.	1.00	Ref.	1.00	Ref.	1.00	Ref.
Number of ANC visits								
Inadequate	**0.56**	**(0.42–0.76)** ^**a**^	**0.75**	**(0.58–0.98)** ^**c**^	**0.76**	**(0.58–0.99)** ^**c**^	**0.72**	**(0.62–0.84)** ^**a**^
Adequate	1.00	Ref.	1.00	Ref.	1.00	Ref.	1.00	Ref.
Smoking								
No	0.45	(0.06–3.50)	1.21	(0.16–9.13)	1.53	(0.17–13.8)	0.90	(0.27–3.05)
Yes	1.00	Ref.	1.00	Ref.	1.00	Ref.	1.00	Ref.
Place of residence								
Urban	1.05	(0.70–1.58)	0.96	(0.64–1.42)	**1.37**	**(1.00–1.87)** ^**c**^	1.18	(0.96–1.45)
Rural	1.00	Ref.	1.00	Ref.	1.00	Ref.	1.00	Ref.
Geographical region								
Northern	1.36	(0.95–1.96)	1.01	(0.70–1.45)	**1.74**	**(1.27–2.39)** ^**b**^	**1.40**	**(1.15–1.70)** ^**b**^
Central	0.98	(0.73–1.33)	0.83	(0.60–1.14)	1.16	(0.87–1.55)	1.00	(0.84–1.19)
Southern	1.00	Ref.	1.00	Ref.	1.00	Ref.	1.00	Ref.

*ANC* Antenatal Care, *BMI* Body Mass Index, *AORs* Adjusted Odds Ratios, *95% CI* 95% Confidence Interval; ^*a*^*P* < 0.0001; ^*b*^*P* < 0.001; ^*c*^*P* < 0.05

All boldface entries are significant as the confidence interval revealed

## Discussion

The aim of this study was to explore the trends of maternal overweight, obesity, and caesarean birth and to determine whether maternal overweight and obesity are risk factors for caesarean birth in Malawi. Studies have linked overweight and obesity to a wide range of unfavourable pregnancy outcomes including maternal and neonatal morbidity and mortalities. [12, 13, 27]. Our findings indicated that maternal obesity is associated with an increased risk of caesarean birth across all the cohort years. After adjustment for potential confounders, compared to women with normal BMI, overweight women were significantly more likely to have caesarean births in 2015/16 and 2004–2015. Similarly, there was an observed increase in the prevalence of caesarean births in 2004–2015.

As with previous findings, [14, 15, 28], we found an increased risk of cesarean birth in overweight and obese women. Although there is limited knowledge about the increased rate of cesarean birth in overweight and obese women, it has been suggested that pregnancy complications such as gestational diabetes, hypertension, increase in maternal pelvic soft tissue, fetal macrosomia, prolonged time of delivery and intrapartum complications might be related to obesity which are known risk factors for caesarian birth [15, 29]. A previous study on maternal obesity and labour complications found that obese women who required induction of labour were associated with increased rates of caesarean birth [30]. Due to the large body volume of obese women, more time may be needed for oxytocin to reach the optimal tissue level. During delivery, feto-placental circulation may be compromised by excess intra-abdominal adipose causing mechanical obstruction of labour and fetal distress prompting the need for caesarean birth [31]. Previous studies in Africa showed that obese women are 87% more likely to have caesarean birth than those who are not [14, 32]. Similarly, elsewhere the risk of cesarean birth was reported to have increased by half in overweight women and two-folds for obese women compared to those with normal BMI [15, 33]. Additionally, maternal obesity is associated with chronic conditions and macrosomic births which may result in cephalopelvic disproportion and prompting the need for caesarean birth [30, 34]. The increased risk for caesarian birth in the Northern Region of Malawi might reflect the small population and the distribution of facilities [35], thus, suggesting that women in the Northern region might have better access to CS services than women in other regions.

There are several limitations to this study that require considerations when interpreting the findings. First, the use of a cross-sectional study design did not allow us to establish temporal relationships. Second, our data are prone to interviewer bias due to social desirability effects. Third, although MDHS data collection instrument was validated to ascertain correct responses from participants, the possibility of recall bias on maternal age, ANC visits, and children’s age may still have occurred, and accordingly, the effects of those covariates might have been either underestimated or overestimated in the study. Fourth, we were unable to adjust all confounding factors such as lifestyle, underlying medical conditions (commodities) and environmental factors because these variables were not included in the MDHS dataset. Finally, the measure of maternal BMI during the survey might have been different from BMI before birth. Generally, women tend to have a high BMI during pregnancy [36] and decrease after birth. Therefore, the strength of association may then possibly be biased towards the null. Further studies using BMI collected during pregnancy may be required to validate our findings. Despite these potential limitations, our study has notable strengths. These data came from a well validated population-based surveillance registry representative of the referent population which enables our results to be generalized to women of reproductive age in Malawi. In addition, our results may help trigger obesity prevention intervention programs specifically and effectively for this population.

## Conclusions

The current study showed a significant association between overweight and obesity and increased risk of cesarean birth. There is a need for effective personal and public health initiatives to reduce weight before pregnancy in Malawian women. Overweight and obese women should be considered high risk, and diagnosing and monitoring their weight status should be a routine prenatal care procedure. Weight management programs should be implemented in the primary healthcare clinics to counsel and promote weight loss before pregnancy in Malawi. Women of reproductive age should be sensitized to maintain a healthy weight with specific prevention programs on socio-cultural factors that will facilitate the adoption of healthy eating behavior. This can enable women with severe pre-pregnancy obesity to safely achieve quite strict targets for normal weight gain in pregnancy, limiting their risk for the caesarian section and other pregnancy complications.

## Abbreviations

ANC: Antenatal Care
aOR: Adjusted Odds Ratio
BMI: Body Mass Index
CI: Confidence Interval
CS: Caesarean Section
DHS: Demographic Health Survey
GEE: Generalized Estimating Equations
MDHS: Malawi Demographic Health Survey
MPHC: Malawi Population and Housing Census
VIF: Variance Inflation Factor
WHO: World Health Organization
